# Evaluation of “Cognitive Behavioral Intervention for Trauma in Schools” (CBITS) in child welfare programs in Germany: study protocol of a randomized controlled trial

**DOI:** 10.1186/s13063-024-08190-x

**Published:** 2024-06-19

**Authors:** Elisa Pfeiffer, Loni Dörrie, Jessica Köksal, Fabienne Krech, Rainer Muche, Jacob Segler, Cedric Sachser

**Affiliations:** 1 Clinic for Child and Adolescent Psychiatry/Psychotherapy, Ulm University, Steinhoevelstr. 1, 89075 Ulm, Germany; 2 Institute of Epidemiology and Medical Biometry, Ulm University, Schwabstr. 13, 89075 Ulm, Germany

**Keywords:** Randomized controlled trial, Trauma, Children and adolescents, Group intervention, CBITS, Child welfare

## Abstract

**Background:**

Children and adolescents growing up in child welfare institutions have been frequently exposed to traumatic events and psychosocial stress and show elevated rates of mental disorders. Yet, there is a lack of empirically supported treatments to provide adequate mental health care for children in care suffering from trauma-related mental disorders such as posttraumatic stress disorder (PTSD), depression, and anxiety. The Cognitive Behavioral Intervention for Trauma in Schools (CBITS) is an evaluated trauma-focused cognitive-behavioral group intervention, which has proven to be effective in reducing symptoms of PTSD, depression, and anxiety for traumatized children in group settings. The trial will evaluate the effectiveness of the CBITS intervention as an outreach treatment compared with an enhanced treatment-as-usual condition (TAU +) within the German mental health and child welfare system.

**Methods:**

In a randomized controlled trial (RCT) involving *N* = 90 children and adolescents, we will compare CBITS with TAU + . Participants between 8 and 16 years, reporting at least one traumatic event and moderate posttraumatic stress symptoms (PTSS), will be randomized within their child welfare institution to either one of the conditions using a CATS-2 severity-stratified block randomization. Assessments will take place at baseline, as well as 4 months and 10 months after baseline. The primary outcome is the severity of PTSS after 4 months. Secondary outcomes are depression, anxiety, irritability/anger, quality of life, and global functioning level.

**Discussion:**

The results of our trial will provide evidence regarding effective treatment options for traumatized children in care, which represent an understudied population with limited access to mental health care. Additionally, it could serve as a blueprint for implementing trauma-focused outreach group treatments for children in care and increase the accessibility to appropriate treatment.

**Trial registration:**

Clinical Trials.gov NCT06038357 D. September 13, 2023.

**Supplementary Information:**

The online version contains supplementary material available at 10.1186/s13063-024-08190-x.

## Background

Children and adolescents in (inpatient) child welfare and care institutions (dt. “Kinder- und Jugendhilfeeinrichtungen”) are probably the group in our society that is or has been exposed most frequently to extreme psychosocial stress and sequential traumatization. A study by Jaritz et al. [1] found that 75% of the children and adolescents in child welfare programs and care institutions in Germany had experienced at least one traumatic experience in their lifetime, while 51% had experienced various different kinds of traumatization. Additionally, crucial psychosocial and biological risk factors for the development of mental health problems (such as premature birth, prenatal exposure to noxious substances, and genetic predispositions) accumulate in this population [2]. As a consequence, mental health problems and, in particular, trauma-related mental disorders are significantly more common in child welfare populations than in the general population [3]. Several studies focusing on adolescents in foster care reported lifetime prevalence rates for posttraumatic stress disorder (PTSD) of 14–16% based on structured diagnostic interviews (e.g., [4]).

Recent meta-analysis [5] and international treatment guidelines [6] highly recommend trauma-focused cognitive behavioral approaches to treat PTSD and comorbid trauma-related disorders such as depression or anxiety disorders in traumatized children and adolescents. The individual treatment “trauma-focused cognitive behavioral therapy” (TF-CBT; [7]) is one of the treatments with the most empirical support worldwide and was found to be effective in German routine clinical care as well [8]. Next to a large body of evidence on the effectiveness of individual treatments, there is growing evidence on the feasibility and efficacy of trauma-focused group treatments. In fact, school- and community-based interventions in a group setting are one way of meeting the high demand for mental health care in vulnerable populations [9]. A recent meta-analysis by Davis et al. [10] examined the effectiveness of group-based interventions in reducing PTSD symptoms in 6–18-year-old children and adolescents. In total, they included 42 studies (*N* = 5998). The researchers found that participants in the group-based intervention had significantly fewer PTSD symptoms and improvement in depressive symptoms compared with control groups (PTSD symptoms: *g* =  − 0.55, CI [− 0.76, − 0.35]). Particularly, cognitive-behavioral interventions were effective in treating PTSD symptoms, especially in children and adolescents who experienced complex trauma (such as war experiences or sexual abuse). These interventions were often delivered by less trained staff or with translated versions of the manual and still demonstrated effectiveness.

One of the most researched trauma-focused cognitive-behavioral group interventions is “Cognitive Behavioral Intervention for Trauma in Schools” (CBITS; [11]), which has been scientifically evaluated and sustainably implemented across many regions in the USA, Australia, China, Japan, and Guyana. So far, the intervention has been evaluated in school settings in the USA and demonstrated promising results in a first pilot-test quasi-experimental study (*N* = 199 children; ages 8–15) in which CBITS was compared with a control condition (PTSD: *d*_pre-post/_CBITS_ = 0.67; *d*_Korr_ = 0.44; depression: *d*_pre-post_CBITS_ = 0.39; *d*_Korr_ = 0.34) [12] and a following RCT (*N* = 126 children; ages 10–12 years) regarding PTSD (reported effect size of 1.08 SDs) and depression (reported effect size of 0.45 SDs) at 3-month follow-up [13]. A field trial after Hurricane Katrina in New Orleans [14] (*N* = 118 children; ages 9–15, 55.9% female) showed significant results in terms of reduction in PTSD and depression among those who participated in CBITS (*n* = 57; *d*_PTSD_ = 0.72; *d*_depression_ = 0.42) and those who participated in trauma-focused cognitive behavioral therapy (TF-CBT) (*n* = 14; *d*_PTSD_ = 1.16; *d*_depression_ = 0.47). Although students who received the interventions improved in both arms of the study, uptake of the intervention was uneven across the groups, with 98% starting CBITS and only 37% beginning TF-CBT at a nearby clinic. This finding highlights the importance of low-threshold accessibility of an intervention as well as the high feasibility of CBITS.

So far, CBITS has not been evaluated in the German mental health system and in child welfare settings. Solely one study by Auslander et al. [15] adapted and implemented “Girls Aspiring toward Independence” (GAIN), a trauma-focused, group-based therapy which is an extended version of CBITS for girls in child welfare. They included *N* = 27 girls (12–18 years old) and randomized them either to an experimental or usual care condition. The results showed, despite the small sample size, significant symptom reductions (PTSS and depression) in the intervention group and lower reductions in the control condition. However, in a subsequent RCT [16], evaluating the intervention with adolescent girls (*N* = 249) in the child welfare system in the USA, PTSD, and depression decreased in both conditions, but the intervention was not superior to usual care. Only in regard to clinical improvement more participants in the intervention condition reported PTSS and depression symptoms under the clinical threshold post-intervention, compared to usual care. They found that the intervention was an acceptable model for the population though.

In sum, despite the results of the studies with (slightly older) girls in child welfare by Wendy Auslander and colleagues, CBITS seems especially appropriate for traumatized children in child welfare due to the (cost-effective) group component, low-threshold accessibility, and because the program focuses on reducing trauma symptoms and providing skills to handle stress and trauma in the future.

There is a shortfall between the mental health needs of the high-risk population in German child welfare programs and the treatment options made available to them. Due to several individual (e.g., self-stigma) and structural (e.g., lack of trained therapists in trauma-focused evidence-based treatments (EBTs), limited coordination between mental health professionals and child welfare staff) barriers, this large number of traumatized children has very little access to evidence-based trauma-focused care. Outreach treatments in which the therapist would travel to individuals, families, or child welfare programs to deliver the treatments have been recommended as this lowers the psychological and practical barriers to treatment [17]. In fact, until today, “Mein Weg” (Engl. “My Way”) for traumatized refugee minors is the only trauma-focused group treatment that has been systematically implemented, evaluated, and disseminated in child welfare programs in Germany [18].

The present study therefore aims at evaluating CBITS in the German mental health care system and systematically implementing it into routine care at child welfare institutions. Within the randomized controlled trial (RCT), trauma-exposed children with at least moderate posttraumatic stress symptoms (PTSS) will be randomized to either CBITS (intervention group) or treatment as usual enhanced (TAU + ; control group) in their child welfare institution stratified by CATS-2 severity. All participants will be assessed at baseline (T0), at 4 months (T1), and 10 months (T2) follow-up to evaluate the effectiveness of the intervention regarding symptom reduction compared to the control condition, on the one hand, and the feasibility of the group intervention within child welfare programs, on the other hand. In sum, not only the delivery of an evidence-based group program, but also the outreach engagement strategy is absolutely novel in the German health care service context. We expect that such a strategy will significantly improve access to EBTs, engagement in therapy, and outcomes of children and adolescents exposed to trauma and consequently help to decrease individual and societal costs of victimization during childhood and adolescence in the long term.

## Methods

### Aims and hypotheses

We aim to evaluate the intervention CBITS for children and adolescents being cared for in German child welfare programs and to systematically implement the intervention into routine care. The objectives are as follows:

1. Primary objective: Evaluate the effectiveness of CBITS compared to TAU + regarding PTSS symptom reduction (primary outcome), anxiety, depression, irritability/anger, quality of life and functional level, and continuance of the child welfare program (secondary outcomes) at 4-month follow-up
2. Investigate potential long-term effects of the treatment in the treatment condition regarding the primary and secondary outcomes at 10-month follow-up
3. Implement CBITS as an outreach intervention into routine mental health care for traumatized children, to evaluate treatment fidelity and treatment completion and investigate different potential individual or structural factors that might have an impact on the implementation
4. To assess the readiness of child welfare programs to collaborate with mental health services and the role of institutional environments for developmental trajectories

We expect CBITS to be superior to TAU+ regarding mental health outcomes, quality of life, functional impairment, and continuance of the child welfare program. Based on the current literature, we propose the following hypothesis regarding our primary objective:

H1: The CBITS intervention is superior in reducing PTSS compared with the control condition (TAU+) post-intervention.

The following secondary hypotheses will be tested in an exploratory manner:

H2a: The CBITS intervention is superior in reducing depression, anxiety, and irritability/anger compared with the control condition post-intervention.

H2b: The CBITS intervention is superior regarding improvements in quality of life, functional impairment, and continuance of the child welfare program compared with the control condition post-intervention.

H3: Symptom reduction within the CBITS intervention group is stable until 10-month follow-up post-intervention.

### Trial design

**Fig. 1:**
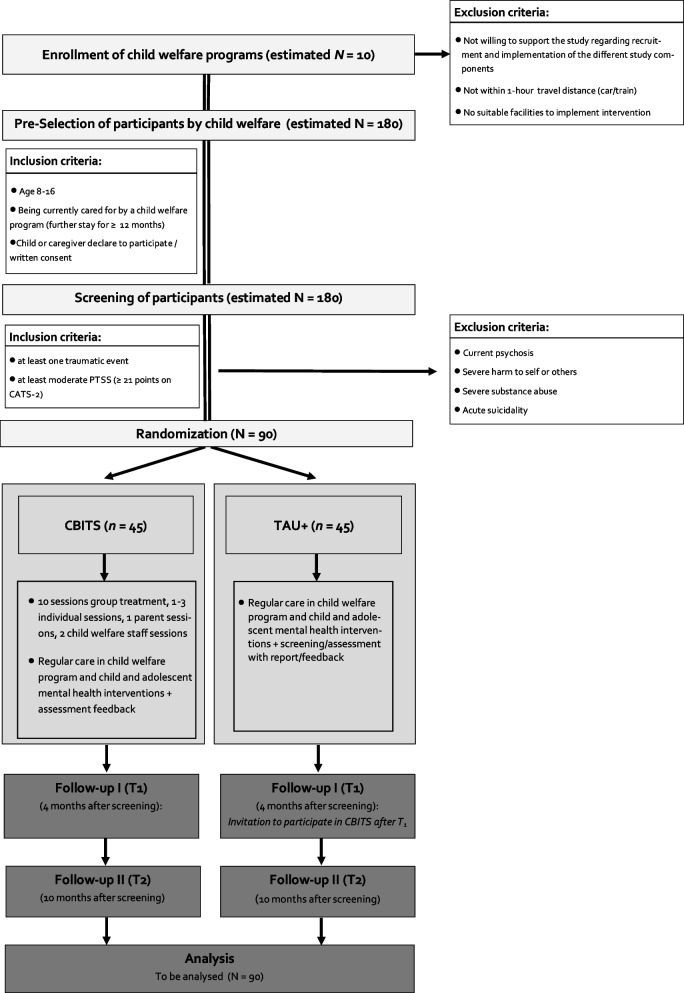
Participant flow through the study

The current study is a randomized controlled trial with two active conditions (CBITS vs. TAU +) and three measurement time points. An independent institution (Institute of Epidemiology and Medical Biometry, Ulm University) will perform the randomization. The randomization will be conducted within each child welfare program. Hence, half of the participants within the program will be in the CBITS condition and the other half will be in the TAU + condition. Randomization will be done in the group of registered participants as CATS-2-stratified block randomization using the randomization software ROM [25]. Participants will be randomized in either CBITS or TAU + after their agreement to participate in the study and after the screening (T0) of participants of the respective facility is completed. The participants and their CATS-2 severity score will be reported as a list and will be sent to the Institute of Epidemiology and Medical Biometry for randomization. See Fig. 1 for participant flow through the study.

The study protocol was written in accordance with the SPIRIT 2013 statement (Standard Protocol Items: Recommendations for Interventional Trials; for the SPIRIT Checklist see Additional file 1, for SPIRIT Table see Table 1).

**Table 1:**
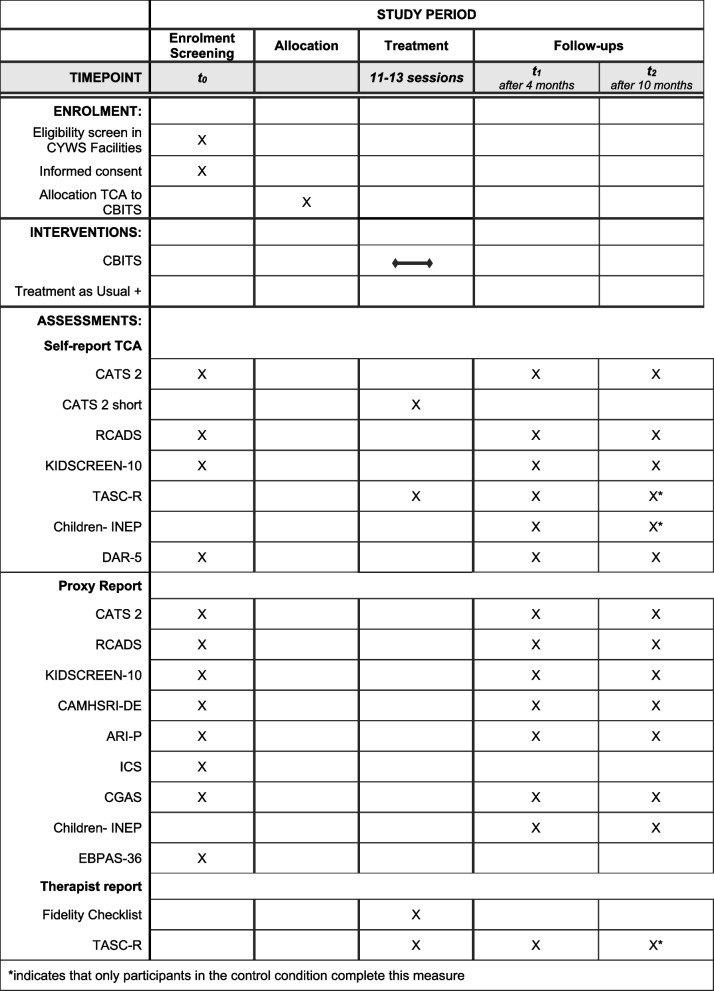
SPIRIT table

### Study setting and recruitment

As this is an outreach study, assessment and intervention will take place outside of the clinical setting: the child welfare facilities in which the children and adolescents live. Researchers from a university clinic will be involved in instructing screenings and implementing the intervention (workshop, case consultation).

Recruitment of participants will be conducted by specifically trained child welfare staff (these so-called child welfare project coordinators receive a workshop from the project staff) in the respective programs. We will use the following recruitment strategies: (1) The general staff in the child welfare program will be invited to a digital information session on the study with all its components (e.g., assessment, treatment, collaboration with study centers). (2) The child welfare project coordinator and trained child welfare staff will organize information meetings with potential study participants to inform them about the project before the first assessment. (3) Information material on the project (poster, flyers, or postcards) will be made available for children and youth in the child welfare program.

To recruit therapists, we will build collaborations with certified training institutes. The following recruitment strategies will be implemented to identify therapists for the CBITS implementation: (1) We will inform the leaders in the study centers about the study and discuss potential recruitment strategies with them. (2) We will establish cooperation agreements with the study centers in which each of them agrees to help recruit at least eight therapists for the study. In this agreement, we will specify the benefits for the therapists for their education program (e.g., CBITS group therapy sessions and case consultations will be part of their regular education) on the one hand and financial incentives on the other hand. We will further specify the (financial) benefits of the center for recruiting therapists. (3) Information material on the project (poster, flyers, or postcards) will be made available for therapists at the cooperating centers. (4) We will offer digital information meetings for interested therapists on the study, the intervention, and their tasks.

General additional recruitment efforts will include a study website, press interviews, distribution of flyers, publications in newspapers and journals, and presentations at national and international scientific conferences.

### Participants and eligibility criteria

#### Criteria for participants

The inclusion criteria for participants are (1) ages 8–16 years, because this age range reflects the age range in the CBITS evaluation studies, and this age range is specified in the CBITS manual and toolkit; (2) having experienced at least one traumatic event (based on DSM-5 or ICD-10/ICD-11 criteria) as CBITS is designed for children and adolescents with a trauma history; and (3) at least moderate PTSS (at least 21 points on the CATS-2) as this is recommended in the intervention manual. Participants do not have to fulfill the PTSD criteria as the manual does not name this a pre-condition for participation. Additionally, there is large evidence that also youth with subthreshold PTSS show high functional impairment but respond very well to trauma-focused interventions; (4) being currently cared for by a child welfare program (safe and stable living conditions), planned further stay in the program for at least 12 months in order to complete the CBITS intervention and 10-month follow-up; and (5) willingness and informed assent/consent of the participant as well as informed consent of the caregiver/legal guardian to participate in the study (sufficient motivation for group intervention and compliance with the study design).

In order to assure a high external validity of the study, we will apply only very few exclusion criteria: (1) current psychosis for safety reasons and because, in this case, another intervention has priority (same explanation for 2–4); (2) severe harm to self or others; (3) severe substance disorder; (4) acute suicidality; and (5) severe mental retardation as there is a certain necessity of sufficient cognitive abilities to benefit from CBITS, to recall trauma memories and to create a trauma narrative.

#### Criteria for therapists

In order to be able to complete the CBITS training program and to deliver the intervention, potential study therapists at the participating study centers in Ulm, Bochum, Mannheim, and Marburg have to meet the following inclusion criteria: (1) have sufficient English language skills to complete web-training and follow the 3-day training workshop as well as case consultations; (2) be enrolled in the psychotherapy training program at one of our study centers for at least another 6 months; (3) at least 1 year of psychotherapy training to have sufficient prior cognitive behavioral therapy (CBT) experience to conduct the intervention; and (4) complete written informed consent.

### Sample size

Based on the literature and our planned analyses on the effectiveness of CBITS for children, we expect a moderate effect size of *d* = 0.7 for the CBITS intervention. An attempt was made to estimate the likely effect size based on longitudinal studies reporting on children and adolescents living in the child welfare system: In our own trial of unaccompanied refugee minors, we found an effect size of *d* = 0.15 for routine care in child welfare programs [18]. A Swiss study investigating externalizing behavior problems in institutionalized adolescents over the course of 1 year found effects of *d*_girls_ = 0.28 and *d*_boys_ = 0.24 [19]. Another study investigating emotional and behavioral symptom trajectories of children in long-term out-of-home care in an English local authority found that the largest trajectories were chronic symptom profiles, in which young people were rated in the abnormal range from their first year in care and remained in this range (based on the descriptive statistics reported we calculated *d* = 0.02–0.1 for emotional problems [20]). Based on these studies, we assume a mean effect size of *d* = 0.1 for routine care for children in child welfare programs. As children and adolescents randomized to TAU + will also receive a report with a treatment indication, we assume that a small proportion in TAU + condition (10–20%) will find their way to local service providers. For those receiving psychotherapeutic treatment from local service providers, we assume a mean effect size of *d* = 1.0 based on therapy as usual conditions in other trials (e.g., [21]). In sum, based on the before-mentioned literature on routine care in child welfare systems and the assumption that a small proportion will receive treatment at local service providers, we assume an overall mean effect size of *d* = 0.3 for the TAU + condition. Following the expected effect size of *d* = 0.7 for CBITS and *d* = 0.3 for TAU + , we assume a controlled effect size of *d* = 0.4 between conditions at 4-month follow-up (T1) in favor of CBITS on the primary measure of PTSS (CATS-2). A power analysis conducted with G*Power for the within-(time)-between-(condition)-interaction requiring a power of 0.80 and *α* = 0.05 (two-tailed), a correlation of *r* = 0.3 among replicated measures (based on our previous own RCTs [18, 22]) estimates a sample size of *N* = 72 (*n* = 36 per condition) to detect the controlled effect size of *d* = 0.4 for the primary outcome between conditions. With an assumed dropout rate of about 20% across both conditions, an oversampling of *n* = 18 participants is needed, resulting in an overall sample of *N* = 90 participants (*n* = 45 per condition; *n* = 22–23 per participating site).

### Procedure

#### Participants

The child welfare project coordinator and child welfare staff will be informed about participant inclusion criteria beforehand to ensure that all participants willing to participate are eligible. They will also receive information on the general study protocol, the intervention, and their tasks in the project. The written informed consent forms provided by the legal guardian will be assessed by the child welfare project coordinator before baseline. The written informed consent forms of the participants will be assessed directly by the study team during baseline assessment.

The total duration for one participant in the trial from screening to the 10-month follow-up is estimated for about 12 months. The individual participant and the respective caregiver will participate in three study visits at the respective child welfare facility (baseline assessment (T0), post-treatment/4-month follow-up assessment (T1), and 10-month follow-up assessment (T2)). All participants will complete self-report questionnaires via the platform REDCap and caregivers (child welfare staff) will complete respective proxy reports via paper–pencil or REDCap, at each assessment time point. At the end of every assessment, the participant will receive a brief feedback on the PTSS, depression, and anxiety scores by the researchers, and a brief written report will be sent to each participant via mail several days after each assessment. The inclusion and exclusion criteria will be assessed at T0. After the inclusion of all participants in the child welfare program, the participants will be randomized to either CBITS or TAU + by sending a list to the randomization center. The outcome of the randomization will be communicated via phone to the child welfare project coordinator and also sent via mail in an official letter. The time span between randomization and the first treatment session should be no longer than 4 weeks. After randomization, the study staff will match a trained therapist to the intervention group and help organize the intervention sessions both with the therapist and the child welfare project coordinator (who will inform participants and caregivers). In each intervention session, the therapist will assess all participants with the CATS-2 ICD-11 PTSD short version via paper–pencil format, collect the questionnaires, and submit them to the study staff. The therapists and participants will complete an alliance rating after the first session and after the last session of the CBITS intervention and the therapists complete a fidelity rating after each CBITS session. The fidelity ratings and CATS-2 symptom monitoring data will be added to an online survey (www. unipark.de) by the therapist after each session.

### Therapists

The therapists who are enrolled in the study will complete their questionnaires via paper–pencil, which will be sent to them via mail. After the therapists are enrolled in the project, they will receive a token for the CBITS web training. Once they completed the web training (confirmation by submitting their certificate to the study staff per email), they will be invited to the 3-day basic training. After the basic training, they will be contacted once there is a child welfare program ready to start the CBITS intervention at their facilities. Lastly, the therapists will be enrolled in a biweekly case consultation group. After all the training and implementation steps, the therapists will receive a certificate to be an approved CBITS therapist.

### Intervention

In the following, both active conditions, CBITS and TAU + , will be described in more detail.

### CBITS

The CBITS program is a skills-based trauma group and individual intervention, which uses evidence-based cognitive-behavioral techniques (e.g., psychoeducation, relaxation, social problem solving, cognitive restructuring, and exposure) and is designed to be delivered by mental health professionals. The theoretical underpinnings are based on cognitive behavioral theory regarding anxiety and trauma. The program consists of ten 45-min group sessions (about six to eight students/participants per group), one to three individual sessions, two child welfare staff psychoeducational sessions, and one parent/caregiver session. For this study, we will specifically train and supervise study therapists to deliver the intervention within child welfare programs. For children in child welfare, CBITS offers several advantages over traditional community-based, individual-level treatment models. First, with its focus on trauma, CBITS has been shown to influence PTSS, depression, anxiety, and school behavior, making it an ideal means to target the diverse problems that children in child welfare face. Second, unlike traditional community mental health services, CBITS is most often provided outside of the clinical setting, making it possible to keep youth in place to receive the program rather than pulling them away for clinic-based treatment. Third, the group format of CBITS broadens the reach of the intervention, making it possible to serve more youth than a traditional individual-based therapy model. From an ethical standpoint, it seems highly appropriate to provide this vulnerable population with a helpful trauma-focused group intervention.

### Implementation of CBITS in the study

Regarding dose (number and length of sessions) and mode (group and individual sessions) of application, this study will closely stick to the evaluated manual and guidelines as there were no known safety issues in the above-described RCTs. In order to implement CBITS, study therapists at the four study centers receive training in CBITS from the CBITS developers at the RAND research cooperation (https://www.rand.org/about.html). We will develop and offer a CBITS training package consisting of a multimedia online training (https://cbitsprogram.org/public-training), an instructor-led 3-day workshop on the intervention, and case consultations on ongoing group interventions via digital conference. After each session, therapists will complete a fidelity checklist (online survey) which will be submitted to the study staff who forward it to the case consultants. The CBITS manual and workbook materials were translated into German in October 2023 and the permission for distribution was granted in November 2023.

### Treatment as usual + 

In the control condition (TAU +), child welfare programs will follow their usual procedures (i.e., routine care of child welfare, referral to medical practitioners and psychotherapists, and handling of prescribed medication, referral to inpatient treatments in case of risk to self and others) which reflects treatment as usual in child welfare programs and the mental health care system in Germany. Additionally, participants in the control condition will receive the same baseline assessment and reporting of screening results as participants in the treatment condition after each assessment. With each report, participants receive a feedback on their scores in the questionnaires and a recommendation (e.g., psychotherapy) that they can follow with guidance from the child welfare staff. Additional treatments will be closely monitored by an adapted version of CAMHSRI-DE [23] in both conditions. After the first follow-up assessment (T_1_; 4 months), all participants in the control condition who still report clinically relevant PTSS (at least 21 points on the CATS-2) and would like to receive the group treatment will be offered participation in CBITS.

### Measures

All self-report measures of the participants and proxy report of the caregivers at the child welfare programs will be assessed via REDCap. Weekly symptom monitoring during CBITS and therapist measures will be assessed via an online survey.

### Primary outcome

The Child and Adolescent Trauma Screen Version 2 (CATS-2) [24] is a questionnaire to screen for potentially traumatic experiences and 20 DSM-5 and ICD-11 symptoms of PTSD in children and adolescents. The measure has been developed by an international expert group led by the Ulm University (applicant and co-applicant of this proposal). The DSM-5 PTSD total score (*α* = 0.89), the ICD-11 PTSD total score (*α* = 0.67), and the ICD-11 CPTSD total score (*α* = 0.83) have proven acceptable to excellent reliability. This CATS-2 version also includes items for measuring ICD-11 Complex PTSD and offers a short version for symptom monitoring during treatment with only 7 items, which will be used in this study for weekly symptom monitoring in the CBITS condition.

### Secondary outcomes

All secondary outcomes will be evaluated at the 4-month and 10-month follow-up. Anxiety and Depression will be measured using the “Revised Child Anxiety and Depression Scale” (RCADS) [25]. The RCADS is a 47-item, youth self-report questionnaire developed to measure DSM-IV relevant symptoms of anxiety disorders (separation anxiety disorder, social phobia, generalized anxiety disorder, panic disorder) as well as symptoms of obsessive–compulsive disorder and depression in children (*α* = 0.73–0.96) [26].

Anger and aggression will be measured via the “Dimensions of Anger Reactions” (DAR-5) [28] rating scale. It is a self-report measure which assesses anger frequency, intensity, duration, and aggression with five items. It has shown a very good internal reliability (*α* = 0.86) [29].

The KIDSCREEN-10 is a generic questionnaire used to measure health-related quality of life via ten items (*α* = 0.77–0.89) [27].

### Additional measures

Socio-demographic data will be assessed with a 12-item questionnaire including the following demographic information: gender, age, duration in child welfare (overall), duration in current child welfare program, number of different placements, contact to family members, relationship status of parents, educational level, and ethnic and social background.

Therapeutic Alliance will be measured via the “Therapeutic Alliance Scale for Children—Revised” (TASC-r). The questionnaire assesses the working alliance between therapists and participants with 12 items, which has shown excellent reliability for participants (*α* = 0.88–0.91) [30]. To assess the Group Alliance, 12 additional items were created based on the original items adapted to the group setting.

The “Inventory for the Assessment of Negative Effects of Psychotherapy for Children and Adolescents” (Children-INEP) consists of 18 items which assess unwanted side effects of the therapy [31].

### Proxy measures

The following measures will be detailed by the respective caregiver of each participant:

The CATS-2 caregiver version [24] which comprises the same items as the CATS self-report with a reference to the child/adolescent (“has your child…”) will be used to measure proxy reported PTSS. The DSM-5 PTSD total score (*α* = 0.91), the ICD-11 PTSD total score (*α* = 0.79), and the ICD-11 CPTSD total score (*α* = 0.87) have proven acceptable to excellent reliability.

Anxiety and depression will be assessed by the RCADS-P (parent-version) [25]. The 47-item inventory measures DSM-IV-relevant symptoms of anxiety disorders (separation anxiety disorder, social phobia, generalized anxiety disorder, panic disorder) as well as symptoms of obsessive–compulsive disorder and depression in children (*α* = 0.73–0.96) [26].

The KIDSCREEN-10 is a generic questionnaire used to measure health-related quality of life via 10 items (*α* = 0.77–0.89) [27]. The parent version will be used and adapted to the caregiver environment.

Affective Reactivity Index-Parent (ARI-P): The ARI-P [25] is a short seven-item instrument to assess irritability in Children via proxy report. The reliability was proven to be good (*α* = 0.89) with an adequate test–retest reliability (ICC = 0.67, 95% CI [0.14, 0.85] [39].

The “Child and Adolescent Mental Health Service Receipt Inventory” (CAMHSRI-DE) [23] is a questionnaire for evaluating the use of medical, psychosocial, and child welfare services, which consists of eight parts assessing the number of additional treatments and medication for the previous months.

The Children-INEP consists of 18 items to assess unwanted side effects of psychotherapy via caregiver report [31].

The “Evidence-Based Practice Attitude Scale” (EBPAS-36, [32]) is a 36-item questionnaire derived from the EBPAS-50. It assesses the attitudes of mental health providers toward the adoption of evidence-based practice, which has been well-validated.

The “Implementation Climate Scale” (ICS, [33]) measures the degree to which the organizational climate is supportive of the evidence-based practice implementation using 18 items. High internal consistency has been confirmed for the total scale (*α* = 0.91) and for the subscales (*α* = 0.81–0.91).

The “Children’s Global Assessment Scale” (CGAS) is an instrument that assesses the level of global functioning. It has proven good inter-rater reliability (*α* = 0.84) and test–retest reliability (*α* = 0.85) [34]).

Socio-demographic data (age, gender, education, work experience, and level of experience regarding the work with traumatized children) will be assessed with a self-created eight-item questionnaire.

To assess serious adverse events (SAEs), the caregivers will complete a standardized checklist. SAEs will be assessed at baseline and follow-ups and as soon as a SAE occurs. If an incident is to be reported, caregivers fill in a detailed report about the SAE and send it to the study center.

Feedback on the study and intervention will be systematically assessed by participants, care givers, and therapists at 4- and 10-month follow-ups.

### Measures for CBITS therapists

Socio-demographic data will be assessed with a 15-item non-standardized questionnaire (therapists’ gender, age, work experience, education, level of experience with trauma and traumatized children in particular).

The TASC-r [30] will be used to assess the working alliance between the therapist and the children with 12 items (*α* = 0.88–0.91). The instrument was translated into German, and 12 additional items adapted to measure the working alliance between the therapist and the group were added. Another 12 items based on the original ones were created that reflect the working alliance perceived out of the therapist’s perspective.

The Treatment Fidelity Checklist will be completed after each CBITS session to assess whether the specific topics were covered. The checklist was translated into German during the trial preparation phase.

### Methods against bias

Random allocation of participants is ensured by the trial design. Stratification by CATS-2 will ensure comparability for PTSS at baseline (T0). The CBITS training will be highly standardized and implemented by certified intervention trainers, treatment fidelity will be ensured by ongoing case consultations and by session checklists. Compensation of the additional training time as part of the regular training and providing access to an otherwise expensive additional clinical training without extra costs will reduce the selectivity of the therapist population under study. The blinding of the participants and therapists is not possible. Study assessors will not be blinded by condition either, but only self-report measures will be used and not clinician-rated interviews. All outcomes on the participant, caregiver, and therapist levels are psychometrically sound and well-established measures. Primary and secondary outcomes are determined in the study protocol to avoid selective reporting (reporting bias/publication bias). Data records will be kept as up-to-date as possible by means of online symptom monitoring and documentation of treatment sessions. All available follow-up data will be included in the final analyses, thus investigating an intention to treat (ITT) population of participants. The mixed model analysis will take missing data and different numbers of reassessments per participant into account. The trial is preregistered at https://clinicaltrials.gov/, and its results will be published irrespectively of significant results to avoid publication bias. The publication of results will be in line with the CONSORT guidelines.

Important protocol modifications will be communicated to trial registries, journals, regulators, funders, advisory board, and DSMB right away via e-mail and conference calls. Trial participants will be informed by letters.

### Data management and storage

Data from the participants will be directly entered via tablets into a specifically designed database (REDCap). Data collected using the paper–pencil procedure will be afterward entered in the database (REDCap) by a data manager. Incoming data will be continuously monitored and checked for plausibility, quality, and completeness by a data manager. All data will be stored in pseudonymized form in the database. Only authorized and trained study personnel will receive a login-roll in line with their task (e.g., research assistant to start screening, data manager for query management, data entry, data validation, and plausibility checks). For the purpose of long-term storage, the original, pseudonymized data (after database lock) will be stored at Ulm University.

An anonymized participant-level data will be made available on request to scientific colleagues after publication of the results.

### Statistical analysis

The primary outcome (CATS-2 self-report) will be analyzed using mixed effects models (MEM) with fixed effects of group (CBITS vs. TAU +) and time (baseline T0 versus 4-month follow-up T1) as well as their interaction (*α* = 0.05). Based on the longitudinal design of the data structure, data will be nested by participants; and repeated measures will be modeled using an unstructured covariance matrix. Since MEMs can handle missing data within the longitudinal data structure, the analyses will be performed with the ITT sample including all randomized participants (*N* = 90). We will use exploratory MEMs for the secondary outcomes. Additionally, per-protocol analyses will be performed for all outcomes.

We could identify several factors that might be related to dropout in children who receive trauma-focused treatment such as number of traumatic events (e.g., [35]). Hence, we will run a sensitivity analysis with the following variables: child age and gender, pre-treatment PTSS level, number of traumatic events, and type of treatment. In more detail, we will run the analysis while controlling for these potential covariates, repeat the analysis not controlling for the factors, and check for differences in the results. When finding the differences, we will use the model with the covariates to allow for making the assumption of missing at random for an unbiased effect estimate. Accounting for non-compliance is not necessary for the ITT analyses. For the per-protocol analyses, we will adjust for possible covariates for non-compliance in order to assume strong ignorability plausible. Effect sizes will be calculated by Cohen’s *d* [36]. A significance level of *α* = 0.05 will be used. Exploratory subgroup analyses for known PTSS outcome predictors (e.g., age, sex, baseline PTSS) will be performed. Safety: We will compare the frequency of SAEs between conditions using the *χ*^2^ tests (*α* = 0.05). No interim analyses will be performed on the main outcomes.

### Ethical considerations, safety, and monitoring

The study was planned and will be conducted in accordance with the International Council for Harmonization Guideline for Good Clinical Practice [37]. The trial is registered under ClinicalTrials.Gov (https://clinicaltrials.gov; registration number NCT06038357, date September 13, 2023). It has been approved by the ethics review board of Ulm University (November 27, 2023; Nr. 267/23 – FSt/Sta). All participants will be informed about the study in oral and written form, with details of the trial’s procedures, risks, costs, confidentiality, data storage, and about the right to discontinue participation at any time without giving any reasons. Participants will be free to continue any other treatment in case they quit the research program. Written informed consent will be obtained from all study therapists, caregivers, and participants (as well as from their parents/legal guardians) before study inclusion.

In general, treatment and the associated processes of change and diagnostic procedures may be evaluated as stressful by the participant [38]. Therefore, symptoms may temporarily worsen. In the context of CBITS, the development of a trauma narrative can be experienced as stressful as it involves direct confrontation with the traumatic experience and avoided stimuli. However, participants can directly benefit from successful treatment, and long-term symptom reduction can be achieved. Even for the TAU + group who will only have the possibility to receive CBITS after T_1_, we do not expect any safety issues as they can participate in any other medical or psychotherapeutic treatment during the trial. Study safety will be ensured by monitoring for the incidence of serious adverse events (SAEs; e.g., suicide attempts, unplanned hospitalizations, occurrence of life-threatening conditions) at all assessments. All such incidents and other aspects of study safety will be regularly reported to an independent Data and Safety Monitoring Board (DSMB), which offers advice on protocol changes in the event of such incidences, or even on discontinuation of the trial. In addition, study therapists participating in the study will report SAEs of a study participant they treat as soon as they gain knowledge about it (within two working days). Any SAEs occurring before, after, or between screening appointments will be reported by the child welfare project coordinators at the child welfare programs immediately (the latest within two working days). The study protocol will also include several standard operations of procedure (SOP) which will guide the study staff and therapists through the safety study procedures.

The study offers different incentives/compensations to the participants, the child welfare programs, and therapists for participation in the study.

An international advisory board with leading experts in the field of PTSD treatment for children and adolescents was established. Both the advisory board and the DSMB will hold online regular meetings with the study team and provide continuous advice during the trial.

The quality and completeness of all data will be continuously monitored by an independent data manager located at Ulm University, who will ensure that the trial is conducted and data are generated, documented, and reported, all in compliance with the study protocol, GCP guidelines, and other applicable regulatory requirements.

## Discussion

This study will be the first RCT on the effectiveness and implementation of a trauma-focused group intervention for traumatized children and adolescents in child welfare programs, which will be implemented outside of the clinical setting. As the included study participants constitute an especially vulnerable population for developing trauma-related disorders on the one hand, and oftentimes only have limited access to trauma-focused EBTs in Germany on the other hand, this study might provide crucial insights on how to improve their short- and long-term mental health. The study also aims to report on different implementation aspects of trauma-focused (group) interventions outside of routine clinical care and to potentially inspire future scientific efforts in documenting factors, facilitating the implementation of these interventions in settings in which children and adolescents already spend most of their time (schools, daycare, child welfare, etc.) and feel more comfortable in than in a clinical setting.

### Dissemination

The findings of this trial will be disseminated both within academia (i.e., scientific papers, presentations at conferences) and outside of academia (i.e., on a societal level). As there is currently no evidence for an outreach group treatment delivered by therapists in child welfare programs in Germany, one major dissemination goal of this study is to inform policymakers on the potential effectiveness and benefits of establishing such interventions long-term. The results of this trial will also be communicated to participants, therapists, and child welfare staff included in the trial and to larger therapist organizations and child welfare networks in Germany. With an open-access publication of the German version of the manual and other CBITS materials, we want to motivate other practitioners to consider offering the intervention after the study. The sustainable implementation of the CBITS intervention will enable trained therapists to implement trauma-informed care not only for study participants but also for non-participants. This trial will hopefully be able to model future translations of outreaching EBTs into regular clinical training programs and child welfare programs across Germany.

### Strengths and limitations

This study has several strengths: (1) By recruiting participants in several different child welfare programs in four different regions in Germany, we will endeavor to recruit a sample that is as representative as possible. (2) The project is aware of the challenges in the child welfare programs (e.g., staff turnover) and will seek to determine the facilities’ readiness to collaborate with mental health services. (3) By randomizing the participants within the child welfare programs and using the control condition TAU + where we document all (mental) health care offers to the participants, we aim to furnish reliable evidence of the actual care for children and adolescents cared for by the child welfare system in Germany. (4) This study claims a high external validity that represents the natural mental health and child welfare context in which children and adolescents in Germany normally reside. (5) The mixed-methods approach on assessment of feedback to the intervention by participants and therapists will allow us a high level of participant involvement.

Possible limitations to the trial are as follows: (1) Some measures will have to be translated to German and are therefore not validated in this language version. (2) The assessment only includes self-report questionnaires and no clinical interviews to ensure a high feasibility of the trial. We will not, therefore, be able to report on clinical diagnoses. (3) We believe that the child welfare programs participating in the trial will show a high motivation to improve mental health care for traumatized children and adolescents who cannot live with their (biological) parents that is probably beyond average. Additionally, therapists willing to participate may be particularly interested in providing EBTs, which could result in a selection bias.

### Trial status

This study protocol is version 1 from November 30, 2023. Recruitment of participants in child welfare programs began on May 1, 2023, and will be completed in January 2025. Recruitment of child welfare programs also began on May 1, 2023, and will be completed in December 2024. Lastly, recruitment of therapists began on June 12, 2023, and will be completed in December 2024 as well. At the time of manuscript submission (December 22, 2023), the study recruitment had started, and the first inclusion of a patient is planned for February 2024.

Up until manuscript submission, ten child welfare programs in the areas around the cities Ulm, Bochum, Mannheim, and Marburg were identified as recruitment centers. We already established cooperation agreements with several child welfare programs for the baseline screening.

### Supplementary Information

Supplementary Material 1.

## Data Availability

The datasets supporting the conclusions of this article are available upon request from the authors.

## Abbreviations

ARI-P: Affective Reactivity Index-Parent
CATS-2: Child and Adolescent Trauma Screen Version 2
CAMHSRI-DE: Child and Adolescent Mental Health Service Receipt Inventory Deutsch
CBITS: Cognitive Behavioral Intervention for Trauma in Schools
CBT: Cognitive behavioral therapy
CGAS: Children’s Global Assessment Scale
Children-INEP: Inventory for the Assessment of Negative Effects of Psychotherapy for Children and Adolescents
EBT: Evidence-based treatment
DAR-5: Dimensions of Anger Reaction
DSM-5: Diagnostic and Statistical Manual of Mental Disorders, 5th edition
EBPAS-36: Evidence-Based Practice Attitude Scale
ICD-10: International Classification of Diseases
ICS: Implementation Climate Scale
ProQOL: Professional Quality of Life
PTSD: Posttraumatic stress disorder
PTSS: Posttraumatic stress symptoms
RCADS: Revised Child Anxiety and Depression Scale
RCADS-P: Revised Child Anxiety and Depression Scale Parent version
RCT: Randomized controlled trial
SAEs: Serious adverse events
SOP: Standard operating procedure
TASC-r: Therapeutic Alliance Scale for Children-Revised
TF-CBT: Trauma-focused cognitive behavioral therapy
